# LRRK2 Inhibition by PF06447475 Antagonist Modulates Early Neuronal Damage after Spinal Cord Trauma

**DOI:** 10.3390/antiox11091634

**Published:** 2022-08-23

**Authors:** Alessia Filippone, Deborah Mannino, Laura Cucinotta, Irene Paterniti, Emanuela Esposito, Michela Campolo

**Affiliations:** Department of Chemical, Biological, Pharmaceutical and Environmental Sciences, University of Messina, Viale Ferdinando Stagno D’Alcontres, 31-98166 Messina, Italy

**Keywords:** neuroinflammation, spinal cord injury, cytokines, oxidative stress

## Abstract

Spinal cord injury (SCI) is a devastating event followed by neurodegeneration, activation of the inflammatory cascade, and immune system. The leucine-rich-repeat kinase 2 (LRRK2) is a gene associated with Parkinson’s disease (PD), moreover, its kinase activity was found to be upregulated after instigated inflammation of the central nervous system (CNS). Here, we aimed to investigate the PF06447475 (abbreviated as PF-475) role as a pharmacological LRRK2 antagonist by counteracting pathological consequences of spinal cord trauma. The in vivo model of SCI was induced by extradural compression of the spinal cord, then mice were treated with PF0-475 (2.5–5 and 10 mg/kg i.p) 1 and 6 h after SCI. We found that PF-475 treatments at the higher doses (5 and 10 mg/kg) showed a great ability to significantly reduce the degree of spinal cord tissue injury, glycogen accumulation, and demyelination of neurons associated with trauma. Furthermore, oxidative stress and cytokines expression levels, including interleukins (IL-1, IL-6, IL-10, and 12), interferon-γ (IFN-γ), and tumor necrosis factor-α (TNF-α), secreted and released after trauma were decreased by LRRK2 antagonist treatments. Our results suggest that the correlations between LRRK2 and inflammation of the CNS exist and that LRRK2 activity targeting could have direct effects on the intervention of neuroinflammatory disorders.

## 1. Introduction

Spinal cord injury (SCI) is a devastating trauma compromising mature central nervous system (CNS) neurons that fail to regenerate after injury, leading to permanent neurological consequences such as pain, disability, and loss of neuron functionality [1]. It has been reported to affect over two million people worldwide with a higher incidence in young men than women [2]. In SCI, after the in situ mechanical and irreversible lesion, associated with ischemia, blood spinal cord barrier (BSCB) disruption, and cell necrosis, the second injury occurs that consists of resident glial cells recruitment followed by maturation of T and B cells as part of the immune adaptive system, together with the activation of the inflammatory cascade associated to cytokines release [3]. Given these, our work provides deep insight into the biochemical inhibition of the protein leucine-rich repeat kinase 2 (LRRK2), linked to familial and sporadic cases of PD and to the pathways regulating inflammation of the CNS. LRRK2 is a large (2527 amino acids) multidomain protein with two catalytic domains: a Ras of complex (ROC) GTPase domain that is able to bind GTP and hydrolyze it, and a kinase domain that uses a series of Rab GTPases as substrates [4]. It is widely expressed in many tissues including the liver, lung, circulating immune cells, and in the brain where its function is not fully understood. Targeting LRRK2 in PD represented a decrease in pathological signs of progression such as a reduction in cytokine levels in the brain protecting neurons from neurodegeneration induced by α-synuclein accumulation [5]. Moreover, mounting evidence supports that because of its multiple enzymatic and protein-binding domains, LRRK2 regulates varied pathways other than PD including the autophagy process by mTOR signaling [6], microglia activation, and CD4 and CD8 cell infiltration in cancer and inflammatory bowel disease (IBS) [7,8,9]. Thus, current therapeutic lines being settled for LRRK2-linked neuroinflammation have focused on the use of LRRK2 kinase antagonists [10,11]. Especially parallel synthesis of one of the most brain penetrant antagonists, chemically known as PF06447475 (abbreviated as PF-475), arrived at a potent and selective LRRK2 inhibition optimization that also displays good tolerance with a relatively high dose (65 mg/kg) in a 2-week toxicological assessment [12]. Thus, we examined the role of PF-475 antagonist of LRRK2 in an in vivo model of SCI by highlighting its ability to modulate neuronal metabolic homeostasis and cytokines release in the spinal cord after trauma. 

## 2. Materials and Methods

### 2.1. Materials

PF06447475 (abbreviated as PF-475 throughout the main text) was purchased from MedChemExpress LLC (1 Deer Park Dr, Suite Q, Monmouth Junction, NJ 08852, USA; # HY-12477). All stock solutions were prepared in nonpyrogenic saline (0.9% NaCl; Baxter, Liverpool, UK). Unless otherwise stated, all compounds were obtained from Sigma–Aldrich (Milan, Italy). 

### 2.2. Animals

CD1 male mice (25–30 g; 6–8 weeks of age) were purchased from Envigo (Milan, Italy). The mice were placed in a controlled environment and were fed with standard rodent food and water ad libitum. The animals were housed in stainless steel cages in a room maintained at 22 ± 1 °C with a cycle of 12 h of light and 12 h of dark. The animal study was performed in accordance with Italian regulations on the use of animals (D.M.116192) and Directive legislation (EU) (2010/63/EU) amended by Regulation (EU) 2019/1010. The animal protocol was approved by Italian Committee (n° 399/2019-PR released on 2019).

### 2.3. SCI Procedure

Mice were anesthetized intraperitoneal (i.p.) with xylazine and ketamine (0.16 and 2.6 mg/kg body weight, respectively). Then, a longitudinal incision was made in the midline of the back, exposing the paravertebral muscles [13]. These muscles were dissected away, exposing the T5 to T8 vertebrae. Once the spinal cord was exposed, it was subjected to extradural compression using an aneurysm clip with a closing force of 24 g, for 1 min. During recovery from anesthesia, the mice were placed on a warm heating pad and covered with a warm towel. After 24 h, animals were euthanized, and spinal cord tissues were collected for histological examinations and biochemical analyses. Control mice were only subjected to laminectomy. Moreover, PF-475 treatments at the doses of 2.5–5 and 10 mg/kg were administered 1 and 6 h postinjury since these time points reflect clinically relevant and effective times (therapeutic window) for counteracting acute damage. PF-475 was dissolved in dimethyl sulfoxide (DMSO) and diluted with 0.9% saline to a final concentration of <1% DMSO. 

### 2.4. Experimental Groups

Mice were randomly divided as follows:

- Sham + vehicle: mice were subjected to laminectomy, but the aneurysm clip was not applied; these mice were administered with saline + DMSO intraperitoneally, 1 and 6 h after laminectomy;
- SCI + vehicle: mice were subjected to SCI plus intraperitoneal administration of saline + DMSO;
- SCI + PF-475 2.5 mg/kg: mice were subjected to SCI plus intraperitoneal administration of PF-475 at the dose of 2.5 mg/kg 1 and 6 h after SCI;
- SCI + PF-475 5 mg/kg: mice were subjected to SCI plus intraperitoneal administration of PF-475 at the dose of 5 mg/kg 1 and 6 h after SCI);
- SCI+PF-475 10 mg/kg: mice were subjected to SCI plus intraperitoneal administration of PF-475 at the dose of 10 mg/kg 1 and 6 h after SCI;

The chosen experimental doses were obtained after dose–response preliminary study in our laboratory. The PF-475 route of administration was based on previous in vivo study [14]. Data regarding mice subjected only to laminectomy and administered intraperitoneally with PF-475 at different doses (2.5–5 and 10 mg/kg) are not shown because we observed no significant changes compared to the Sham + vehicle mice group. Mice were divided following simple randomization and partial blinding methods as previously described [15]. Moreover, the authors were blinding while performing all the experiments. The minimum number of mice for every technique was estimated with the statistical test “ANOVA: Fixed effect, omnibus one-way” with G-power software. This statistical test generated a sample size equal to n = 10 mice for each technique.

### 2.5. Basso Mouse Scale Score

The motor function of SCI-injured mice was evaluated for 10 days after injury. Recovery from motor disturbance was graded using the Basso Mouse Scale (BMS) open-field score [16]. The BMS scale consists of 8 points: from 0 (indicating complete paralysis) to 8 (indicating normal hind limb function), and rates locomotion on hind limb function including weight support, stepping ability, coordination, and toe clearance. 

### 2.6. Histological Evaluation

At 24 h after SCI, spinal cord tissue was collected and fixed in 10% (*w*/*v*) PBS-buffered formaldehyde solution at 25 °C for 24 h, dehydrated with graduated ethanol, and embedded in paraffin (obtaining 7 μm thick sections). Hematoxylin and eosin (H&E) staining was performed to investigate histological damage and loss of tissue architecture. After H&E staining, sections were examined by light microscopy using objective lens at 10× and 20× magnification (AxioVision, Zeiss, Milan, Italy). Histological score followed a five-point scale [13] on the basis of the following morphological criteria: (0) no pathological abnormalities; (1) small, scattered areas of axonal swelling, morphologically unremarkable tissue in >75% of the perilesional area; (2) significant damage with normal gross architecture, unremarkable tissue in 50–75% of the perilesional area; (3) significant damage with normal gross architecture, morphologically unremarkable tissue in 25–50% of perilesional area; (4) significant damage and loss of gross architecture in large areas, morphologically unremarkable tissue in 10–25% of perilesional area; (5) complete dissolution of the spinal cord tissue over the entire perilesional area with loss of gross architecture, morphologically unremarkable tissue in <10% of perilesional area. The results from every section of the spinal cord were averaged to obtain a final score (1 to 5) for distinct mice. The results of histological examinations were showed at 10× (100 µm scale bar) and 20× magnifications (50 µm scale bar). 

### 2.7. Luxol Fast Blue (LFB) Staining

To assess the degree of myelination/demyelination process, staining with LFB stain kit (Abcam, Waltham, MA 02453, USA # ab150675) was performed in the deparaffinized sections following manufacturer’s instructions. Briefly, spinal cord sections were incubated in LFB solution at 56 °C O/N, then washed in 95% alcohol. Subsequently, the sections were incubated in lithium carbonate solution and 70% ethyl alcohol and, finally, counterstained in the cresyl violet solution. After dehydration, the sections were assembled with Eukitt (Bio-Optica, Milan, Italy) and observed by light microscopy (AxioVision, Zeiss, Milan, Italy). The slides were analyzed by a pathologist blinded to the treatment groups. Images were taken focusing on the perilesional area of SCI and shown by using objective lens at 10× and 20× magnification.

### 2.8. Periodic Acid Schiff (PAS) Staining

PAS staining was performed according to the manufacturer’s instructions (#04-130802/LBio-Optica, Milan, Italy). Spinal cord sections were analyzed using light microscopy connected to an imaging system software and images were taken by using objective lens at 10× and 20× magnification (AxioVision and Software; Zeiss, Milan, Italy), and the number of glycogen granules was counted focusing on the perilesional area of SCI. The slides were analyzed by a pathologist blinded to the treatment groups.

### 2.9. Enzyme-Linked Immunosorbent Assay (ELISA Kit)

The ELISA kit assay was performed on the protein extract of the spinal cord samples to determine the concentration of two proinflammatory cytokines: interleukin-4 (IL-4) (ab100710) and interferon-γ (IFN-γ ab100689). 

### 2.10. Western Blot Analysis of p-LRRK2, LRRK2, IL-6, IL-10, IL-1β, IL-13, TNF-α, GYS, and p-GSK3-α/β

Western blot analysis allowed to evaluate the expression of p-LRRK2, LRRK2 proteins, and those involved in inflammatory processes such as interleukin-1β (IL-1β), tumor necrosis factor-α (TNF-α), IL-6, IL-13, and IL-10. Western blot analysis was performed as previously described [13]. Membranes were incubated at 4 °C overnight with each of the previously mentioned primary antibodies diluted in milk [17], PBS, and 0.1% Tween-20 (PMT): antiphosphorylated LRRK2 at Serine 935 (p-Ser935) (1:1000, Abcam ab133450); anti-LRRK2 (1:1000, Abcam ab133474); anti-IL-1β (1:500; Santa Cruz Biotechnology sc-32294), anti-TNF-α (1:500; Santa Cruz Biotechnology sc-52746), enzyme glycogen synthase (GYS) (1:500; Santa Cruz Biotechnology, sc 81173), glycogen synthase kinase 3 (GSK-3-α/β) (1:500; Santa Cruz Biotechnology, sc 7291), and phosphorylated GSK-3-α/β (p-GSK-3-α/β) (1:500; Santa Cruz Biotechnology, sc 81496). Finally, membranes were incubated for 1 h at room temperature with a secondary antibody and bands were obtained using a chemiluminescence detection system (ECL) according to the manufacturer’s instructions (Thermo, Waltham, MA, USA). Protein expression was quantified by band densitometry and standardized to GAPDH (anti-GAPDH antibody 1:500; sc-32233; Santa Cruz Biotechnology, Dallas, TX, USA) levels as an internal control. The relative expression of the protein bands was quantified by densitometry with BIORAD ChemiDocTMXRS + software.

### 2.11. Immunofluorescence Analysis of Glial Cells

Sagittal spinal cord sections were processed for immunofluorescence staining as previously described [18,19]. Sections were incubated with antiglial fibrillary acidic protein (anti-GFAP) (1:100, sc-33673, Santa Cruz Biotechnology, Santa Cruz, CA, USA), or anti-ionized calcium binding adaptor molecule 1 (anti-Iba-1) (1:100, sc-32725, Santa Cruz Biotechnology, Santa Cruz, CA, USA) antibodies in a humidified chamber O/N at 37 °C. Sections were then incubated with conjugated antirabbit Alexa Fluor-488 secondary antibody #A32731 (1:1000 in PBS, vol/vol Molecular Probes, Monza, Italy) for 1 h at RT. Nuclei were stained by adding 2 μg/mL 4′,6′-diamidino-2-phenylindole (DAPI; Hoechst, Frankfurt, Germany) in PBS. Sections were observed at 40× magnification using a Leica DM2000 microscope (Leica, Milan, Italy). Contrast and brightness were established by examining the most brightly labelled pixels and applying settings that allowed clear visualization of structural details while keeping the highest pixel intensities close to 250. The same settings were used for all images obtained from the other samples that had been processed in parallel.

### 2.12. Measurements of Reactive Oxygen Species (ROS)

Homogenates of spinal cord were diluted 1:10 in HEPES–Tyrode solution to obtain a concentration of 5 mg tissue/mL. Five μL of 2′,7′-dichlorodihydrofluorescein diacetate were added to 0.45 mL homogenates in a 24-well plate for 30 min at 37 °C as previously described [20]. The plate was read by using a microplate reader. The data were expressed as relative values of the sham group.

### 2.13. Malondialdehyde (MDA) Assay

To detect lipid peroxidation, MDA levels were quantified in spinal cord tissue as previously reported [21]. 

### 2.14. Statistical Analysis

Experimental data are expressed as expressed +/−SD. Data analysis was performed with one-way ANOVA followed by a Bonferroni post hoc test for multiple comparisons. Only a *p*-value less than 0.05 was considered significant.

## 3. Results

### 3.1. Treatment with PF-475 Antagonist Attenuates SCI-Induced Tissue Damage and Restores Neuronal Metabolic Homeostasis by Reducing Glycogen Accumulation

**Figure 1:**
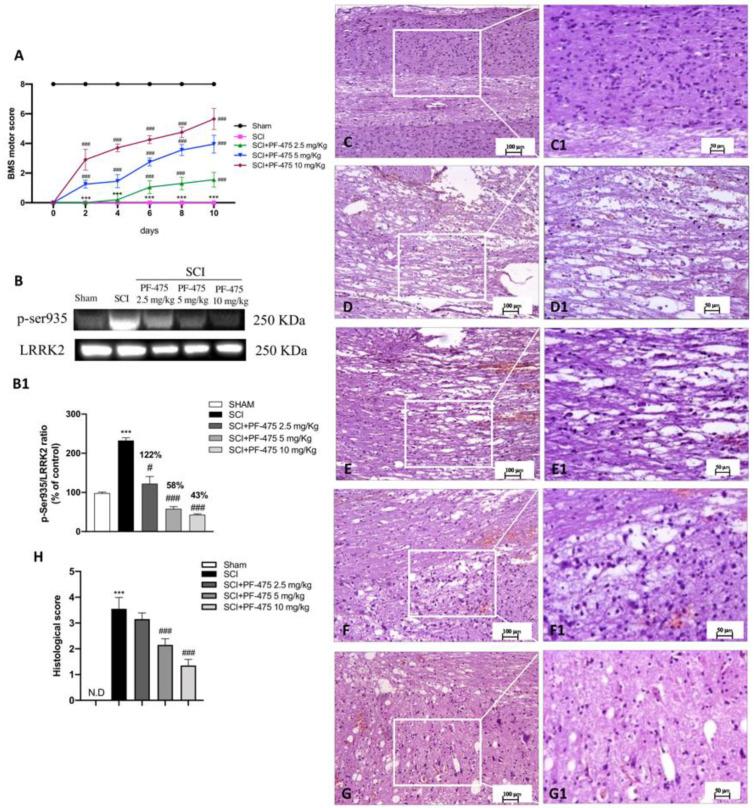
Effects of PF-475 treatments on motor disturbance, phosphorylated LRRK2 at Serine 935 expression, and histological features after SCI. BMS open-field score was performed for 10 days after SCI (**A**). Motor function was not altered in sham mice (black line) compared with SCI- injured mice (pink line), while PF-475 (5 and 10 mg/kg) significantly restored motor functions (blue and purple lines). Representative Western blot of phosphorylated LRRK2 at Serine 935 protein expression level in cytosolic fraction of spinal cord tissues after SCI and PF-475 treatments (**B**,**B1**). The samples used derive from the same experiment and those gel/blots were processed in parallel. Hematoxylin and eosin (**H**,**E**) staining of Sham group (**C**,**C1**) and SCI group ((**D**,**D1**), see histological score (**H**)). PF-475 treatments after SCI: PF-475 2.5 mg/kg ((**E**,**E1**) see histological score (**H**)); PF-475 5 mg/kg ((**F**,**F1**); see histological score (**H**)) and PF-475 10 mg/kg ((**G**,**G1**); see histological score (**H**)). Each data is expressed as SD from ten mice for each group. One-way ANOVA followed by Bonferroni post-test and two-way ANOVA followed by post hoc test for multiple comparisons. (ND) Not detectable. *** *p* < 0.001 vs. sham, # *p* < 0.05 vs. SCI; ### *p* < 0.001 vs. SCI.

To evaluate whether histological damage to the spinal cord after trauma was associated with disturbances of motor function, BMS open-field score was performed for 10 days after SCI (Figure 1A). No significant motor alterations were found in Sham mice (black line in Figure 1A) compared with SCI-injured mice that showed significant deficits in hind limb movement (pink line in Figure 1A). SCI-injured mice treated with PF-475 (2.5 mg/kg, daily) significantly showed a restoration of motor functions starting at 6 days after SCI, while PF-475 administration at the higher doses of 5 and 10 mg/kg significantly restored motor disturbances since day 1 (blue and green lines in Figure 1A).

To clearly demonstrate that PF-475 treatments resulted in LRRK2 kinase activity, we used a phospho-specific antibody against Ser935 phosphorylation site of LRRK2 that is widely used to analyze LRRK2 activity [20]. We observed that PF-475 significantly decreased phosphorylation of LRRK2 at Ser935 (p-LRRK2) indicating pharmacological inhibition of LRRK2 kinase activity. Indeed, a basal expression level of p-LRRK2 was observed in samples from the control group, compared to the significant increase of LRRK2 phosphorylation observed in the SCI group. The higher doses of PF-475 (5 and 10 mg/kg) were shown to be effective in terms of pharmacological inhibition of p-LRRK2 (58% and 43% vs. SCI, respectively), whereas no changes were found for the doses of 1 mg/kg (Figure 1B, see densitometric analysis panel B1, not significant, 122% vs. SCI; F (DFn, DFd) = 40.5, *p* = 0.582, one-way ANOVA method, followed by Bonferroni post hoc test for multiple comparisons).

**Figure 2:**
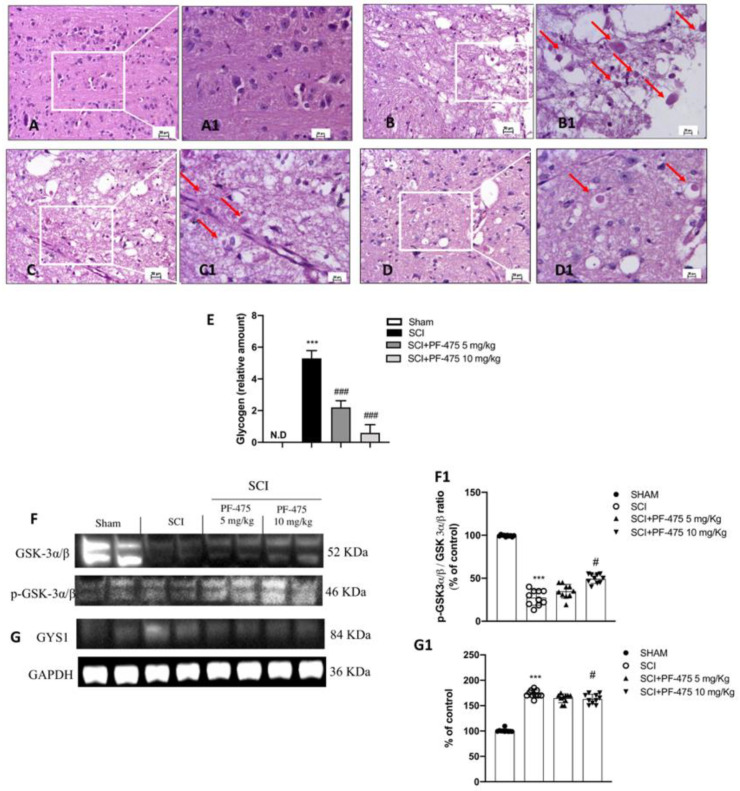
Effects of PF-475 on glycogen metabolism alterations after SCI. Sham group (**A**,**A1**). SCI group (**B**,**B1**). PF-475 treatments after SCI 2.5 and 5 mg/kg ((**C**,**C1**,**D**,**D1**), respectively). Glycogen relative amount (**E**). *** *p* < 0.001 vs. Sham; ### *p* < 0.001 vs. SCI. Cytosolic fractions of spinal cord tissues were used to evaluate glycogen metabolism. Representative Western blots of GSK-3α/β and GYS1 protein expression levels after SCI and PF-475 treatments ((**F**,**G**) and densitometric analysis (**F1**,**G1**), respectively). Each data is expressed as SD from ten mice for each group. One-way ANOVA followed by Bonferroni post-test. (ND) Not detectable. *** *p* < 0.001 vs Sham; # *p* < 0.05 vs SCI. The samples used derive from the same experiment and those gel/blots were processed in parallel.

Histopathological changes after spinal cord trauma were observed in the perilesional area of SCI. We found that SCI-injured mice presented significant tissue alterations associated with an early increase of neutrophil infiltration, edema, and loss of tissue architecture when compared to the sham mice characterized by intact spinal cord tissue (Figure 1C,D, see 20× magnification D1 and C1, respectively, and histological score H). Intraperitoneal administration of PF-475, at the dose of 5 mg/kg and 10 mg/kg (Figure 1F,G, see 20× magnification F1 and G1 and histological score H), showed significant restoration of damaged tissue as well as a reduction in the number of neutrophils and edema. Whereas, treated mice with PF-475 at the dose of 2.5 mg/kg (Figure 1E, see 20× magnification E1 and histological score H) showed no relevant changes compared to the SCI mice; for that reason, we decided not to consider the PF-475 lower dose for further analysis. Moreover, to confirm the histopathological alterations responsible for metabolic homeostasis unbalance after SCI, an accumulation of glycogen was found in the perilesional area of SCI mice related to an alteration of the normal glucose metabolism in the spinal cord. Conversely, the higher number of glycogen granules found in SCI mice compared to the control group (Figure 2A,B, see 20× magnification B1 and A1, respectively, and glycogen amount F) was significantly restored by PF-475 administration at the doses of 5 mg/kg and 10 mg/kg (Figure 2C,D, see 20× magnification C1 and D1, respectively, and glycogen amount E). Next, to confirm the influence of PF-475 antagonist on glycogen metabolism, we evaluated the expression levels of the GYS enzyme, responsible for glycogenesis, and GSK3-α/β, as a key regulator of GYS. Protein expression profiles revealed that GSK3-α/β levels were dramatically decreased after the SCI procedure, while LRRK2 inhibition by PF-475 administration at the higher dose of 10 mg/kg reversed that trend (Figure 2F, see densitometric analysis F1; F (DFn, DFd) = 40.5, *p* = 0.582, one-way ANOVA method, followed by Bonferroni post hoc test for multiple comparisons). By contrast, GYS protein expression levels, found to increase by SCI execution, were attenuated by PF-475 treatments at both doses (Figure 2G, see densitometric analysis G1; F (DFn, DFd) = 40.5, *p* = 0.582, one-way ANOVA method, followed by Bonferroni post hoc test for multiple comparisons). The data obtained give us the chance to consider PF-475 treatment as a reducer of the severity of the lesion after SCI by improving tissue recovery and reducing the metabolic alterations.

### 3.2. LRRK2 Inhibition by PF-475 Treatment Prevents SCI-Induced Demyelination Process 

**Figure 3:**
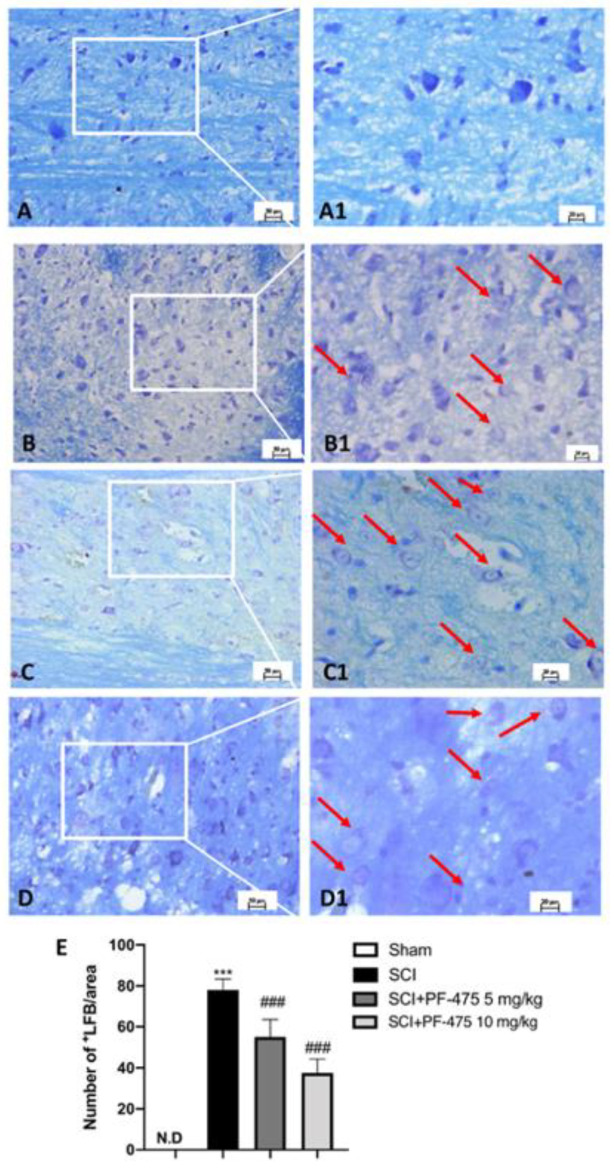
Effects of PF-475 on the myelination process evaluated in spinal cord sections. LFB staining showed healthy neurons (blue) in Sham group (**A**,**A1**) and an accumulation of myelin (purple) in the SCI group (**B**,**B1**). PF-475 treatments (2.5 and 5 mg/kg) after SCI groups reduced the myelin accumulation ((**C**,**C1**,**D**,**D1**) respectively). Number of LFB+ cells over the total LFB area (**E**). Each data is expressed as SD from ten mice for each group. One-way ANOVA followed by Bonferroni post-test. (ND) Not detectable. *** *p* < 0.001 vs. Sham; ### *p* < 0.001 vs. SCI.

Myelin debris and vacuoles were found in the white matter of the spinal cord after trauma and multiple sclerosis because traumatic injury or inflammation leads to promote the axonal degradation process of myelinated fibers [22,23]. For these reasons, by carrying out Luxol Fast Blue (LFB) staining, we investigated the possible effect of LRRK2 antagonist PF-475 by preserving neurons particularly sensitive to SCI. We found that compared to the sham mice, the SCI procedure induced the early stage of the demyelination process in the perilesional area, indicating a high degree of axonal myelin degradation (Figure 3A,B, see 20× magnification A1 and B1, respectively, and number of LFB+ cells E). LFB staining of the spinal white matter of PF-475 (both doses of 5 mg/kg and 10 mg/kg) (Figure 3C,D, see 20× magnification C1 and D1, respectively, and number of LFB+ cells E)-treated mice showed a progressive decrease in the staining of myelin by LFB, suggesting that LRRK2 inhibition prevents myelin loss by exerting a neuroprotective effect after SCI.

### 3.3. PF-475 Treatments Attenuated Glial Cells Activation and Correlated SCI-Induced Oxidative Stress

**Figure 4:**
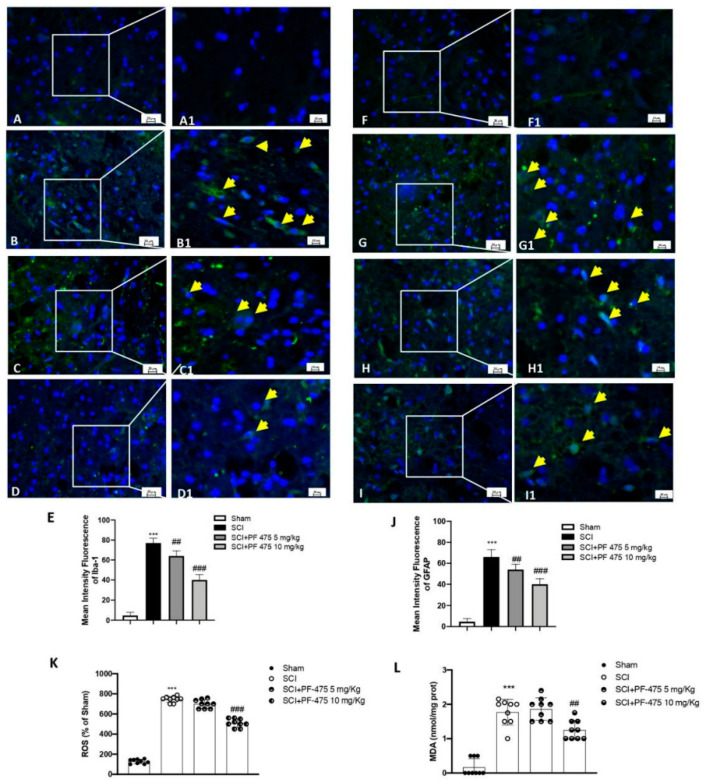
Immunofluorescence analysis of Iba1 and GFAP after SCI. Spinal cord sections were stained with antibodies against Iba1 (green) or GFAP (green) and DAPI to highlight cell nuclei (blue). Sham group ((**A**,**A1**); see mean of intensity fluorescence (**E**)). Iba1 + cells in mice subjected to SCI ((**B**,**B1**); see mean of intensity fluorescence (**E**)). Mice treated with PF-475 at doses of 5 mg/kg ((**C**,**C1**); see mean of intensity fluorescence (**E**)) and 10 mg/kg ((**D**,**D1**); see mean of intensity fluorescence (**E**)). No GFAP + cells showed in. Sham group ((**F**,**F1**); see mean of intensity fluorescence (**J**)). High number of GFAP + cells in SCI group ((**G**,**G1**); see mean of intensity fluorescence (**J**)). PF-475 treatment at a dose of 5 mg/kg ((**H**,**H1**); see mean of intensity fluorescence (**J**)) decrease GFAP+ cells number. PF-475 treatment at a dose of 5 mg/kg ((**I**,**I1**); see mean of intensity fluorescence (**J**)). The content of ROS in spinal cord tissues. (**K**) The concentrations of MDA in spinal cord tissues (**L**). Each data is expressed as SD from ten mice for each group. One-way ANOVA followed by Bonferroni post-test. *** *p* < 0.001 vs. sham and ## *p* < 0.01 and ### *p* < 0.001 vs. SCI.

The acute alterations within the astrocyte and microglia compartments exert main roles in the early onset of spinal cord traumatic injury [16]. Iba-1 and GFAP markers were used to evaluate the active microglia and astrocytes, respectively. A low number of Iba-1+ and GFAP+ cells were found in sham mice, while the SCI procedure significantly increased that number to the higher Iba-1+ and GFAP+ cells (Figure 4A,B, see 100× magnification A1 and B1 and intensity fluorescence E for Iba-1 marker; 4F,G, see 100× magnification F1 and G1 and intensity fluorescence J for GFAP marker). Interestingly, PF-475 administration at the doses of 5 mg/kg and 10 mg/kg significantly decreased the number of both Iba-1+ and GFAP+ cells (Figure 4C,D, see 100× magnification C1 and D1 and intensity fluorescence E for Iba-1 marker; Figure 4H,I, see 100× magnification H1 and I1 and intensity fluorescence J for GFAP marker), suggesting that LRRK2 inhibition by PF-475 reduced microglia and astrocytes activation taking part in the immune innate response of the CNS after trauma. Moreover, microglia recruitment directly triggers SCI-induced reactive oxygen species (ROS) [24]. Thus, in order to evaluate the effects of PF-475 antagonist on SCI-induced oxidative stress, ROS and MDA contents were assessed. As shown in Figure 4K,L, ROS quantity and MDA levels were increased in the SCI model compared to those in the control Sham group, respectively (F (DFn, DFd) = 575, *p* = 0.34, one-way ANOVA method, followed by Bonferroni post hoc test for multiple comparisons) (F (DFn, DFd)= 56.4, *p* = 0.70, one-way ANOVA method, followed by Bonferroni post hoc test for multiple comparisons). Whereas the double administration at 1 and 6 h of PF-475 at the higher dose of 10 mg/kg downregulated MDA levels also attenuating ROS release, suggesting that the SCI-induced oxidative stress was influenced by LRRK2 antagonist. 

### 3.4. Modulation of Inflammatory Cytokines by LRRK2 Inhibition

**Figure 5:**
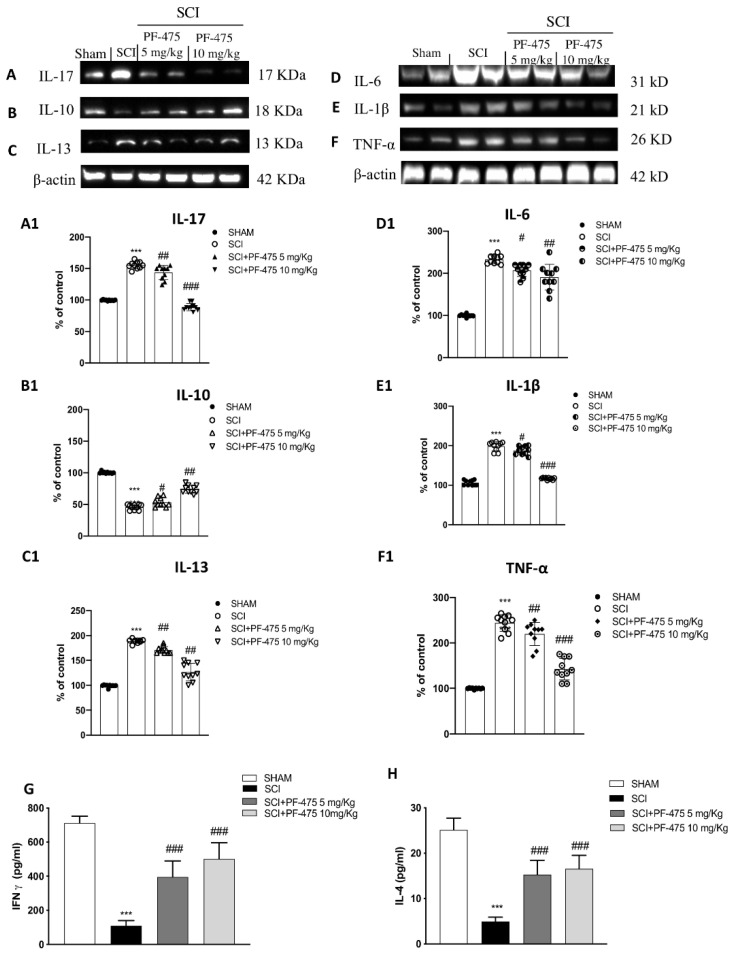
Effects of PF-475 on cytokine expression after SCI. Cytosolic fractions of spinal cord tissues were used to evaluate inflammatory cytokines expression such as IL-17, IL-13, IL-6, IL-1β, and TNF-α, representative blots are shown. Representative blots of inflammatory cytokines such as IL-17, IL-13, IL-6, IL-1β, and TNF-α are shown ((**A**,**C**–**F**), see densitometric analysis (**A1**,**C1**–**F1**). Expression level of the anti-inflammatory cytokine IL-10 (**B**); see densitometric analysis (**B1**). Data represent the means of at least three independent one-way ANOVA experiments followed by post hoc Bonferroni *** *p* < 0.001 vs. Sham; # *p* < 0.05; ## *p* < 0.01 and ### *p* < 0.001 vs. SCI. ELISA method was performed for detection of TNF-α and IL-4 in total protein extract of spinal cord tissues ((**G**,**H**) respectively). Each data is expressed as SD from ten mice for each group. One-way ANOVA followed by Bonferroni post-test. *** *p* < 0.001 vs Sham; ### *p* < 0.001 vs. SCI. The samples used derive from the same experiment and those gels/blots were processed in parallel.

Considering that cytokines are direct mediators of inflammatory response activation after SCI, and are not expressed under physiological conditions [25], we performed Western blot analysis to demonstrate that PF-475, a potent antagonist of LRRK2, was able to significantly reduce the protein expression of many proinflammatory cytokines such as IL-17, IL-13, IL-6, IL-1β, and TNF-α in which increased expression levels was aggravated following spinal cord trauma (Figure 5 blots A, C, D, and F, see densitometric analysis A1, C1, D1, and F1; F (DFn, DFd) = 40.5, *p* = 0.582, one-way ANOVA method, followed by Bonferroni post hoc test for multiple comparisons); F (DFn, DFd) = 40.5, *p* = 0.582, one-way ANOVA method, followed by Bonferroni post hoc test for multiple comparisons); F (DFn, DFd) = 40.5, *p* = 0.582, one-way ANOVA method, followed by Bonferroni post hoc test for multiple comparisons); F (DFn, DFd) = 40.5, *p* = 0.581, one-way ANOVA method, followed by Bonferroni post hoc test for multiple comparisons). Moreover, both PF-475 doses of 5 and 10 mg/kg were able to modulate anti-inflammatory cytokine IL-10 by increasing its protein expression level compared to SCI-injured mice (Figure 5 panel B, see densitometric analysis B1). To confirm PF-475’s ability to promote anti-inflammatory activity, by using specific enzyme-linked assays, we quantified the levels of two other cytokines, such as the IFN-γ and IL-4, reported to have beneficial effects in the CNS [26]. Both levels of IFN-γ and IL-4 were markedly lower in SCI mice compared to control mice, while the expression levels of both cytokines were found increased after PF-475 treatments (both doses of 5 and 10 mg/kg) (Figure 5G,H, respectively), suggesting that PF-475 promotes anti-inflammatory effects of endogenous mediators by attenuation of post-traumatic inflammatory response which probably correlates to the restoration of tissue damage.

## 4. Discussion

Although accumulated evidence shows that modulation of the inflammatory pathway and immune response is crucial in modifying the mechanisms underlying neurodegeneration, neuronal cell death, and related dysfunctions after SCI, the exact mechanism is not fully understood [27]. Certainly, scientific evidence affirms that metabolic imbalances within the spinal cord after injury are responsible for the initiation of motoneurons regression and degeneration, increased activity of some kinases, exacerbating pathological consequences of spinal cord trauma [28]. The discovery of LRRK2 involvement in PD as well as its activity in modulating different inflammatory pathways raised the hope of finding a druggable target for neuroprotective therapies counteracting traumatic consequences in the CNS [29]. Our findings showed that following SCI, the expression level of phosphorylated LRRK2 at the Serine 935 reached a higher level in the spinal cord, confirming that LRRK2 increased kinase activity is simultaneous to varied, correlated events after the traumatic event. By that, the potent antagonistic ability of PF-475 to reduce SCI features including the spinal cord edema, unbalance of glycogen synthesis and degradation, and to reduce early inflammatory cascade activation, which was concomitant with loss of tissue architecture and glycogen accumulation. Extensively, CNS infiltration after SCI by way of inflammatory cells and entry of plasma proteins culminates in the degradation of myelin of neurons of the spinal cord known as the demyelination process, the compromise of neural cell function, and neurobehavioral impairment; and we noticed fewer numbers of cavities in the PF-475 treatment groups filled with degraded myelin in the axons of neurons [30]. The SCI-elicited inflammatory response is a double-edged sword, exerting both damaging and beneficial effects, over time, for the recovery of SCI [31,32,33]. Similar to the neurotoxicity effects of LRRK2 in PD and traumatic brain injury (TBI) [14], our study revealed that protein levels of LRRK2 and cytokines were increased in the injured spinal cord region and peaked at 24 h after SCI. Indeed, inhibition of LRRK2 activity by PF-475 antagonist modulated ILs release in spinal cord tissue after trauma by maintaining lower levels within the acute phase of trauma. In our study, LRRK2 reduced kinase activity by pharmacological inhibition of the phosphorylation process was able to modulate early immune response throughout, slowing down the maturation process of microglia and astrocytes, directly responsible for proinflammatory cytokine release and oxidative stress activation in the CNS after a traumatic event such as SCI. Increased expression of LRRK2 in immune cells in response to proinflammatory signals following traumas has been observed in many immune cell types, strongly suggesting its implication as a specific regulator of the immune response [34]. Although leukocytes, monocytes, and neutrophils have been found in the spinal cord taking part in the immune adaptive response after 2 weeks from SCI in a time-dependent manner, at the early time point of SCI (within 24 h), immune cells are distributed immediately at the lesion epicenter and nearby [35]; therefore, we demonstrated the release of cytokines including IL-4, IL-6, IL-13 and TNF-α and IFN-γ from glial-activated cells while PF-475 was greatly able to potentiate IL-10’s anti-inflammatory effect against the traumatic event of SCI. Additionally, recent studies have reported the toxicity of hyperactivated glial cells around the lesion site [36]. Indeed, we found that, consistently with the results in spinal cord tissues of SCI mice, ROS and MDA levels were decreased by PF-475 antagonist regulating SCI-induced oxidative stress. Despite the poor knowledge about the exact molecular mechanisms involved in LRRK2-mediated neurotoxicity in SCI, PF-475’s heterogenous structure gives it the possibility to strongly influence the upstream functioning in the inflammatory and oxidative stress signaling even in the acute phase after trauma [37]. De facto, the limited pharmacological efficacy of current treatments may be correlated to the matter that drugs such as corticosteroids (i.e., prednisolone) specifically target the activity of neuronal circuitry of the neuroaxis and not that of inflammatory cells that interact with them. Neuroprotection following spinal cord trauma could be also offered by cell-based therapies that provide multiple benefits by transplanting stem cells (MSCs), neural stem cells (NSCs), oligodendrocyte progenitor cells (OPCs), and Schwann cells (SCs) [38] or using biomaterials such as polymers [39]. Our findings suggest that LRRK2 possesses a role in neuroinflammation, a key pathological process that contributes to triggering secondary events after SCI as well as the slowdown in immune system activation. However, there are some limitations worth mentioning in this study. First, the ability of PF-475 by LRRK2 inhibition for counteracting motor disabilities that characterized the primary event after mechanical trauma of SCI in association to ischemia and apoptosis process needs to be evaluated, to confirm the strong role of LRRK2 blocking in modulating pathological consequences of SCI, particularly in the early phases after trauma. Second, the modulation of the neuroinflammation by LRRK2 inhibition should be evaluated by SCI time course, to strongly create a crosslink between the inflammatory response of the CNS and LRRK2 activity.

## 5. Conclusions

In conclusion, this study suggests that LRRK2 will pave the way for a new therapeutic strategy with functional effects for counteracting CNS traumatic events in the early stages. However, a better understanding of LRRK2 activity may be important to determine its inhibition as a therapeutic use, particularly for long-term treatment for CNS pathologies.

## Data Availability

The data that support the findings of this study are available from the corresponding author upon reasonable request.
